# Early post-stroke cognitive impairment and in-hospital predicting factors among stroke survivors in Ethiopia

**DOI:** 10.3389/fneur.2023.1163812

**Published:** 2023-05-22

**Authors:** Gashaw Walle Ayehu, Fitalew Tadele Admasu, Getachew Yideg Yitbarek, Assefa Agegnehu Teshome, Abraham Tsedalu Amare, Daniel Atlaw, Saurab Sharma

**Affiliations:** ^1^Department of Biomedical Sciences, College of Health Sciences, Debre Tabor University, Debre Tabor, Ethiopia; ^2^Department of Adult Health Nursing, College of Health Sciences, Debre Tabor University, Debre Tabor, Ethiopia; ^3^Department of Biomedical Sciences, Goba Referral Hospital, Madda Walabu University, Goba, Oromia, Ethiopia; ^4^School of Health Sciences, Faculty of Medicine and Health, University of New South Wales, Sydney, NSW, Australia; ^5^Centre for Pain IMPACT, Neuroscience Research Australia (NeuRA), Sydney, NSW, Australia

**Keywords:** predicting factors, stroke survivors, Northwest Ethiopia, post-stroke cognitive impairment (PSCI), in hospital

## Abstract

**Background:**

In low-and middle-income countries, post-stroke cognitive impairment (PSCI) is the least investigated stroke complication that clinically is given little attention. Finding patients who are at high risk of having cognitive problems after a stroke could allow targeted follow-up and help with prognosis discussions, which would then contribute to improved treatment outcomes. The main aim of this study was to determine the incidence and predictors of PSCI among stroke survivors in Northwest Ethiopia.

**Methods:**

The study was a multicenter prospective cohort study. The study participants were 403 stroke survivors who were alive on follow-up after 3 months of stroke onset at the neurology department of three hospitals in Northwest Ethiopia. To investigate the link between the outcome and the explanatory variables, analyses of bivariable and logistic multivariable regression were performed. A value of p of 0.05 or less was regarded as statistically significant, and data were presented as odds ratios and 95% confidence intervals.

**Results:**

The mean age of the participants was 61.3 years (SD = 0.7), 56% were females, the mean time from symptom onset to hospital arrival was 46 h (SD = 3.32), and the mean National Institute of Health Stroke Scale (NIHSS) score at admission was 14.79 (SD = 0.25). PSCI was observed in 122 patients (30.3%) after 90 days of stroke onset, that is, 83 (20.6%) of female and 39 (9.7%) of male stroke survivors. The result of multivariable logistic regression analysis revealed PSCI was independently associated with age (adjusted OR = 1.04, 95% CI = 1.061–1.981), women (AOR = 1.390, 95% CI = 1.221–2.690), admission modified Rankin scale (mRS) (AOR = 1.629, 95% CI = 1.381–2.037), moderate Glasgow coma scale (GCS) score (AOR = 1.149, 95% CI = 1.402–3.281), and poor GCS score (AOR = 1.632, 95% CI = 1.610–4.361) and stage one (AOR = 1.428, 95% CI = 1.198–2.922) and stage two hypertension (AOR = 1.255, 95% CI = 1.107–2.609).

**Conclusion:**

Nearly one-third of stroke survivors developed PSCI. Moreover, further research is needed with a larger sample size, showing a time trend and longer follow-up duration.

## Introduction

Poststroke cognitive impairment (PSCI) is the failure of any cognitive function following a stroke, including executive function, memory, language, visuospatial ability, visuoconstructional ability, or global cognitive function (1). Owing to the intricacy of the neuronal networks concerned with cortical processes, the ischemic or hemorrhagic stroke occurring in a specific vascular distribution and damaging a neuroanatomic site often impairs more than one cognitive function (2). Despite the brain’s capacity to adjust for tissue loss, which can lead to cognitive improvement, the majority of patients make little progress and experience long-term cognitive impairment (3). The incidence of PSCI has been increasing due to increasing global trends in aging populations and declining stroke mortality resulting in dramatically higher healthcare costs (4, 5).

After stroke, some individuals may completely regain their physical abilities, while ongoing cognitive impairment disrupts the lives of others with PSCI (6). The mechanism of PSCI remains unclear given heterogeneities in cognitive diagnostic criteria, neurocognitive assessment tools, intervals between cognitive evaluation and stroke onset, prestrike cognitive status, and population characteristics among different studies (2, 7–9).

PSCI is the neglected and least studied complication of stroke in low-and middle-income countries. Identifying patients at high risk of persisting cognitive issues following stroke could allow for targeted follow-up and assist discussions around prognosis, therefore, contributing towards improved treatment outcomes. In addition, there is still a need for more information about the incidence and predictors of PSCI. Therefore, the main aims of this study were to identify the incidence and predictors of PSCI among stroke survivors in Northwest Ethiopia.

## Methods

This study report is aligned with the Strengthening the Reporting of Observational Studies in Epidemiology (STROBE) reporting guidelines (10).

### Study design and setting

**Figure 1:**
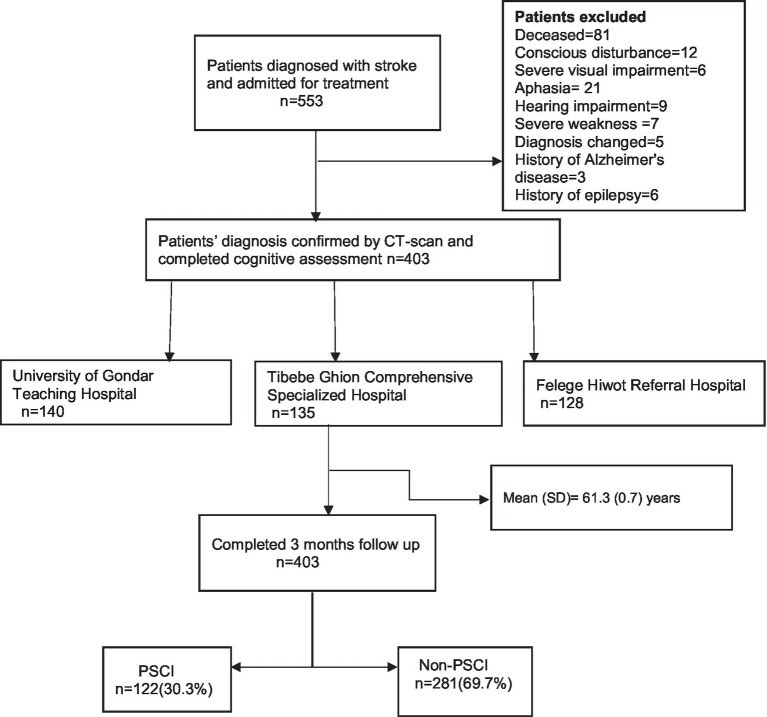
PRISMA flow diagram of participants included in the study.

The study was a multicenter prospective cohort study. This cohort is part of an ongoing project, and data from this cohort has been used previously (11, 12). Data were collected from stroke survivors who were on follow-up at the neurology department of the three hospitals (Figure 1). The sample data were collected from the following hospitals: University of Gondar Teaching Hospital located in Gondar, Tibebe Ghion Comprehensive Specialized Hospital, and Felege Hiwot Referral Hospitals located in Bahir Dar, Northwest Ethiopia, from December 2020 to June 2021.

### Participants

The study population was all stroke survivors who were alive after 3 months of stroke onset. The study participants were 403 stroke survivors who were admitted for in-hospital treatment and on follow-up after completion of admission care at neurology clinics of the hospitals. The inclusion criteria were specified to include all adult (age ≥ 18 years) stroke survivors who were admitted to the hospital and whose diagnosis was confirmed by a CT scan. The study excluded patients who were unable to communicate verbally or had communication problems that could interfere with the performance of cognitive testing such as a history of Alzheimer’s disease, Parkinson’s disease, dementia, epilepsy, head trauma, meningoencephalitis, delirium, motoric disorder on the dominant side, visual impairment, auditory impairment, persistent decreased level of consciousness, and existing neurological and psychiatric disorders.

### Study variables

#### Dependent variable

Post-stroke cognitive impairment (PSCI) which was assessed using the Amharic version of the Montreal cognitive assessment (MoCA). MoCA was translated into Amharic using the forward–backward translation methods. The Amharic version was found to be reliable and valid for Amharic-speaking older adults in Ethiopia (13). “The total score on MoCA ranges from 0 to 30. A score less than 26 indicates cognitive impairment while a MoCA score of 26 or more indicates the normal cognitive function” (14). The questionnaire was assessed by a psychiatrist and a neurologist for clarity and completeness before pre-testing. Pre-testing was done on 5% (15) of the study participants before the start of the actual study at Debre Tabor Comprehensive Specialized Hospital. All the necessary modifications and adjustments were done before implementing the main study.

#### Independent variables

Independent variables included sociodemographic characteristics, behavioral and lifestyle factors, and clinical parameters.

The sociodemographic questions included age, sex, residence, education, occupation, and marital status. Residence is classified as urban and rural when the participant lives in the area for at least 6 months. Education status is further classified as no formal education when the individual is unable to read and write or does not attend formal education, as informal education able to read and write when the individual is able to read and write learned informally like from religious education, as primary education when the participant attends formal education until grade 8, secondary education when the participant attends formal education from grade 9–12, and tertiary education when the individual attends formal education at least diploma and above. Occupation was identified as a housewife when the females take care of the in-house activities, as a farmer when the individual means of livelihood is farming and animal grazing, and as a government employee when the individual means of income is permanent or contractual employment by the government, as a merchant when the means of income is from trade, as a daily laborer when the individual means of livelihood is from daily employment temporarily, and as retired when an individual has served private or government organization until retirement age and means of income is from the pension. Marital status is classified as married when couples declared as married by religion or municipality and if they live in the same house for at least 6 months, unmarried if the participant is single, divorced when the participant was previously married and separated from their partner, and widowed when the participant loses partner by death.

The behavioral factors included alcohol use, cigarette smoking, fruit, and vegetable intake, and physical activity levels. Alcohol use was classified as being a current drinker when patients consumed ≥2 drinks/day in the case of men and ≥ 1 drink in the case of women on average and previous drinkers/past drinkers as those who have stopped drinking for more than 1 year. Cigarette smoking was classified as being current smokers if men smoked two cigarettes per day or if women smoked one per day on average for 1 year. Past/former smokers were those who abstained from smoking for more than a year. Fruit and vegetable eating was defined as eating one time day per week when all meals per day include fruit and vegetables which will be further converted to fruit and vegetable eating per week.

“Physical activity level (PAL) is the measure of planned or incidental activity, including mode or type of activity, frequency of performing an activity, duration of performing an activity, and intensity of performing an activity finally classified as extremely inactive (PAL < 1.4), sedentary (PAL = 1.4–1.69), moderately active (PAL = 1.70–1.99), vigorously active (PAL = 2–2.40), and extremely active (PAL > 2.40).”

The CT scan pattern of stroke type, site of ischemia and hemorrhage, presence of comorbidities, time measured in hours from symptom onset to hospital arrival and from hospital arrival to initiation of treatment, admission blood pressure, “Glasgow Coma Scale (GCS) score having three components, eye-opening, verbal, and motor response scored out of 15 further classified as poor (score of ≤8), moderate (score of 9–12), and good (score of 13–15).” “Modified Rankin Scale (mRS) scores range from zero to six (0 = no symptom, 1 = no disability, 2 = slight disability, 3 = moderate disability, 4 = moderately severe disability, 5 = severe disability, and 6 = dead) where a higher score means worse outcome. National Institute of Health Stroke Scale (NIHSS) scores range from 0 to 42 (0 = no stroke, 1–4 = minor stroke, 5–15 = moderate stroke, 15–20 = moderately severe stroke, and 21–42 = severe stroke)” where a higher score means worse stroke. mRS and NIHSS scores were both measured at admission and discharge.

### Data collection procedure and quality assurance

The high response rate was attained due to data collection conducted by trained physicians and psychiatrists as they were able to explain the objective and relevance of the research. In addition, patients were informed about the result of the first phase of the project which built trust.

Data collection on cognitive function was carried out by one psychiatrist in each hospital with training in data collection while data on the other variables were collected by internal medicine residents (graduating residents) in each hospital.

### Data processing and analysis

The collected data were coded, manually checked, and entered into the data management system by using Epi info version 7. The data were cleaned before analysis, and then, they were exported to STATA version 16 and checked for missing responses. There were no missing responses. Descriptive statistics and numerical summary measures were presented using frequency distribution tables. To investigate the link between the dependent and the independent variables, analyses of bivariable logistic regression were performed to select eligible variables, and then multivariable logistic regression was performed. Multicollinearity was checked using the variance inflation factor (VIF) which was below 10. Data were analyzed using odds ratios (ORs) and a 95% confidence interval was reported. A *p*-value of 0.05 or less was regarded as statistically significant.

## Ethical approval and consent of the participant

The study was performed under the ethical standards of the Helsinki Declaration, Ethical clearance was obtained from the Ethical Review Committee of the College of Health Science, Debre Tabor University, with the reference number CHS/3238/2013 E.C. Informed written consent was obtained from each selected patient; when the patients are not able to give informed written consent, it was obtained from a close relative or caregiver. Neither the case records nor the data extracted were used for any other purpose. The confidentiality and privacy of patients were assured throughout by removing identifiers from data collection tools using different codes.

## Results

### Sociodemographic and baseline characteristics of study participants

The study population comprised 403 stroke survivors at 3 months, with a response rate of 100%. The participants in the study had a mean age of 61.3 years (SD = 0.7), 56% of the participants were women, the mean time from symptom onset to hospital arrival was 46 h (SD = 3.32), and the mean NIHSS score at admission was 14.79 (SD = 0.25). The baseline characteristics of the study participants are shown in Table 1.

**Table 1:** Sociodemographic, behavioral, and baseline clinical profile of stroke survivors in Northwest Ethiopia, 2021.

Variables		PSCI *N* (%)	Non-PSCI *N* (%)	Total *N* (%)
Sex	Male	39 (9.7)	140 (34.7)	179 (44.4)
	Female	83 (20.6)	141 (34.9)	224 (55.6)
Age	20–29	1 (0.2)	5 (1.24)	6 (1.5)
	30–39	2 (0.5)	14 (3.5)	16 (4.0)
	40–49	8 (2.0)	33 (8.2)	41 (10.2)
	50–59	16 (4.0)	79 (19.6)	95 (23.6)
	60–69	42 (10.4)	73 (18.1)	115 (28.5)
	70–79	41 (10.2)	64 (15.9)	105 (26.0)
	80–89	12 (3.0)	13 (3.2)	25 (6.2)
Residence	Urban	30 (7.4)	99 (24.6)	129 (32.0)
	Rural	92 (22.8)	182 (45.16)	274 (70.0)
Education	No formal education	92 (22.8)	177 (43.9)	269 (66.7)
	Able to read and write	12 (3.0)	43 (10.7)	55 (13.6)
	Primary and secondary	10 (2.5)	39 (9.7)	49 (12.2)
	Tertiary +	8 (1.98)	22 (5.4)	30 (7.4)
Occupation	Housewife	78 (19.3)	131 (32.5)	209 (51.9)
	Farmer	31 (7.7)	89 (22.1)	120 (29.8)
	Government employee	6 (1.5)	18 (4.5)	24 (6.0)
	Merchant	4 (1.00)	26 (6.4)	30 (7.4)
	Retired	3 (0.7)	17 (4.2)	20 (5.0)
Marital status	Married	94 (23.3)	235 (58.3)	329 (81.6)
	Widowed	16 (4.0)	13 (3.2)	29 (7.2)
	Divorced	10 (2.5)	20 (5.0)	30 (7.4)
	Unmarried	2 (0.5)	13 (77.7)	15 (3.7)
Alcohol use	Non-drinker	72 (17.9)	163 (40.4)	235 (58.3)
	Past drinker	37 (9.2)	87 (21.6)	124 (30.8)
	Current drinker	13 (3.2)	31 (7.7)	44 (10.9)
Cigarette smoking	Non-smoker	117 (29.0)	272 (67.5)	389 (96.5)
	Past smoker	5 (1.2)	9 (2.2)	14 (3.5)
Fruit and vegetable eating	<2 times per week	119 (29.5)	264 (65.5)	383 (95.0)
	3–4 times per week	3 (0.7)	17 (4.2)	20 (5.0)
Physical activity level	Extremely inactive	4 (1.0)	16 (4.0)	20 (5.0)
	Sedentary	23 (5.7)	52 (12.9)	75 (18.6)
	Moderately active	87 (21.6)	182 (45.2)	269 (66.7)
	Vigorously active	3 (0.7)	7 (1.7)	10 (2.5)
	Extremely active	5 (1.2)	24 (5.9)	29 (7.2)
Comorbidity	No comorbidity	57 (14.1)	163 (40.4)	220 (54.6)
	Hypertension	35 (8.7)	69 (17.1)	104 (25.8)
	Previous stroke	10 (2.5)	4 (1.0)	14 (3.5)
	Congestive heart failure	10 (2.5)	20 (5.0)	30 (7.4)
	Diabetes Mellitus	5 (1.2)	10 (2.5)	15 (3.7)
	Hyperlipidemia	3 (0.7)	7 (1.73)	10 (2.5)
	Atrial Fibrillation	2 (0.2)	8 (2.0)	10 (2.5)
Type of stroke	Ischemic stroke	78 (19.3)	195 (48.4)	273 (67.7)
	Hemorrhagic stroke	44 (10.9)	86 (21.3)	130 (32.2)
Type of hemorrhage	Parenchymal	28 (6.9)	57 (14.1)	85 (21.1)
	Ventricular with subarachnoid extension	12 (2.97)	23 (5.70)	35 (8.7)
	Parenchymal and ventricular	2 (0.5)	3 (0.7)	5 (1.2)
	Parenchymal with ventricular and subarachnoid extension	1 (0.2)	4 (1.0)	5 (1.2)
Site of hemorrhage	Ventricular	17 (4.2)	33 (8.2)	50 (12.4)
	Left cerebral	15 (3.7)	35 (8.7)	50 (12.4)
	Right cerebral	10 (2.5)	10 (2.5)	20 (5.0)
	Subcortical gray matter	2 (0.5)	3 (0.7)	5 (1.2)
	Multi-focal	0	5 (1.2)	5 (1.2)
Site of Ischemia	Left cerebral	31 (7.7)	83 (20.6)	114 (28.3)
	Right cerebral	28 (6.9)	77 (19.1)	105 (26.0)
	Subcortical gray matter	12 (3.0)	22 (5.4)	34 (8.4)
	Muti-focal	5 (1.2)	10 (2.5)	15 (3.7)
	Brain stem	2 (0.2)	3 (0.7)	5 (1.2)
Time from symptom onset to hospital arrival	≤24 h	72 (17.9)	192 (47.6)	264 (65.5)
	25–48 h	19 (4.71)	31 (7.7)	50 (12.4)
	>48 h	31 (7.7)	58 (14.4)	89 (22.1)
Time from Hospital arrival to initiation of treatment	≤3 h	99 (24.5)	214 (53.1)	313 (77.7)
	4–12 h	21 (5.2)	59 (14.6)	80 (19.8)
	13–24 h	1 (0.2)	4 (1.0)	5 (1.2)
	>24 h	1 (0.2)	4 (1.0)	5 (1.2)
Admission GCS score category	Poor	15 (3.7)	15 (3.7)	30 (7.4)
	Moderate	49 (12.2)	116 (28.7)	165 (40.9)
	Good	58 (14.4)	150 (37.2)	208 (51.6)
Admission mRS score	No disability	1 (0.2)	4 (1.0)	5 (1.2)
	Slight disability	2 (0.5)	13 (3.2)	15 (3.7)
	Moderate disability	75 (18.61)	208 (51.6)	283 (70.2)
	Moderately severe disability	44 (10.9)	56 (13.9)	100 (24.8)
	Severe disability	0	0	0
Admission NIHSS score	Minor stroke	3 (0.7)	12 (3.0)	15 (3.7)
	Moderate stroke	46 (11.4)	132 (32.7)	178 (44.2)
	Moderately severe stroke	55 (13.6)	115 (28.5)	170 (42.2)
	Severe stroke	18 (4.4)	22 (5.4)	40 (9.9)
Discharge mRS score	No disability	2 (0.5)	3 (0.7)	5 (1.2)
	Slight disability	28 (7.0)	80 (19.8)	108 (26.8)
	Moderate disability	52 (12.9)	148 (36.7)	200 (49.6)
	Moderately severe disability	39 (9.7)	46 (11.4)	85 (21.1)
	Severe disability	−0.2	4 (1.0)	5 (1.2)
Discharge NIHSS score	Minor stroke	2 (0.49)	7 (1.7)	9 (2.2)
	Moderate stroke	65 (16.13)	189 (46.9)	254 (63.0)
	Moderately severe stroke	40 (9.92)	75 (18.6)	115 (28.5)
	Severe stroke	15 (3.72)	10 (2.5)	25 (6.2)

SD, standard deviation; mRS, modified Rankin Scale; NIHSS, National Institute of Health Stroke Scale; GCS, Glasgow Coma Scale.

### Post-stroke cognitive impairment and its predicting factors

Of the 403 stroke survivors, PSCI was observed in 122 (30.3%) after 3 months of stroke onset. PSCI was found in 83 (20.6%) female and 39 (9.7%) male stroke survivors. The mean MoCA score of the study participants was 26.13 out of 30. The result of multivariable logistic regression analysis revealed that PSCI was independently associated with age, sex, admission mRS score, admission GCS score, and admission systolic blood pressure. Stroke survivors’ increase in age by 1 year (AOR = 1.04, 95% CI = 1.061–1.981) is associated with 1.04 times increased risk of developing PSCI. PSCI was 1.39 times more common among female stroke survivors (AOR = 1.390, 95% CI = 1.221–2.690) than male stroke survivors. Furthermore, the odds of developing PSCI among stroke patients with one score higher in admission mRS score (AOR = 1.629, 95% CI = 1.381–2.037) results in a 62.9% increase in risk compared to one score lower patients. Moreover, the incidence of PSCI was 1.15 and 1.63 times more common in stroke survivors with moderate GCS scores (AOR = 1.149, 95% CI = 1.402–3.281) and poor GCS scores (AOR = 1.632, 95% CI = 1.610–4.361) compared to those with good GCS scores, respectively. The odds of PSCI were 1.2 to 1.4 times higher in people with stage 1 and stage 2 hypertension compared to normotensive individuals during admission (Table 2).

**Table 2:** Predictors of PSCI among stroke survivor patients, Northwest Ethiopia, 2021.

Variables	PSCI	Non-PSCI	COR (95% CI)	AOR (95% CI)
Age	122	281	1.037 (1.054–1.980)***	1.04 (1.061–1.981)***
Sex				
Male	39	140	1	1
Female	83	141	1.473 (1.302–2.739)***	1.390 (1.221–2.690)***
Residence				
Urban	30	99	1	1
Rural	92	182	0.599 (0.371–0.968)*	0.844 (0.482–1.478)
Comorbidity				
No	57	118	1	1
Yes	65	163	0.634 (0.414–0.973)*	0.793 (0.480–1.308)
Admission mRS score	122	281	1.514 (1.341–2.775)***	1.629 (1.381–2.037)**
Admission NIHSS score	122	281	1.960 (1.921–3.983)*	1.024 (0.962–1.091)
Admission GCS score				
Good	15	16	1	1
Moderate	49	115	2.586 (1.188–5.625)*	1.149 (1.402–3.281)*
Poor	58	150	2.586 (1.074–5.214)*	1.632 (1.610–4.361)*
Admission Systolic blood pressure				
Normotensive			1	1
Pre-hypertensive			1.612 (0.258–3.447)	1.564 (0.214–3.484)
Stage 1 hypertension			1.330 (1.162–2.672)**	1.428 (1.198–2.922)*
Stage 2 hypertension			1.239 (1.110–2.521)***	1.255 (1.107–2.609)**
Stage 3 hypertension			1.583 (0.252–2.346)	1.579 (0.235–2.426)
Discharge mRS score			1.719 (1.541–2.956)*	1.915 (0.629–2.331)

*** significant at 0.001 level; ** significant at 0.01 level; *significant at 0.05 level; COR, crude odds ratio; AOR, adjusted odds ratio.

## Discussion

In this first evaluation of PSCI in post-stroke survivors in Ethiopia, we found that PSCI was present in 30.3% of stroke survivors. The mean MoCA score of the study participants was 26.13 out of 30. The result of multivariable logistic regression analysis revealed PSCI was independently associated with age, sex, admission mRS score, admission GCS score, and admission systolic blood pressure.

The occurrence of PSCI in this study is lower than the results found in a pooled estimate from a meta-analysis (44%) (9). The previous studies have reported the incidence of PSCI as 59% in Norway (16), 47% in France (8), 37 to 81% in China (17), 48% in Japan (18), 67% in the United States (19), and 68% in Indonesia (20). The incidence in our study is higher than in an earlier investigation from China (27%) (21). The discrepancy could be attributable to variability in the cognitive evaluation tool used between the studies (MoCA, ACE-R, and MMSE), treatment approach inconsistency across countries, the cutoff point for diagnosis, the size of samples across studies, or the changes in the inclusion criteria between the studies. Moreover, age difference in stroke patients, mode of treatment as admitted patients tend to receive the maximum functional rehabilitation care, and timing of the cognitive assessment may affect variation in incidence of PSCI.

We also found age and sex variation in the incidence of PSCI, older age was associated with an increased risk of PSCI when compared to earlier research from France (8), the United States (15), and China (4). One probable explanation for this is that aging increases vulnerability to neuronal toxicity. Other potential reasons are the rapid increase in median age for stroke incidence and stroke survival rates (22–25). Similarly, in line with a study in the United States (26), we found that females were more likely to have PSCI. This may be accounted for by the fact that sex variances in estradiol hormone expression influence stroke severity which is related to cognitive impairment (27, 28). Moreover, education, occupation, and income level of women affect symptom identification, affording treatment which may interfere with treatment outcomes and affect the cognitive level. In addition, pregnancy complications, contraceptives, and postmenopause hormonal and body changes may modulate stroke severity with a subsequent effect on cognitive outcome. Additionally, the fact that men and women had a similar frequency of stroke might suggest that sex differences in stroke severity, stroke type, stroke location, or other stroke characteristics may explain the sex differences in post-stroke cognitive decline.

We also found that moderate and poor GCS and mRS scores at hospital admission were associated with PSCI in agreement with the preceding studies (15, 29). Both of these ratings are correlated with the severity, size, and duration of the brain damage.

Finally, for participants with higher systolic blood pressure, the odds of PSCI were higher compared to normotensive individuals in accordance with prior studies conducted in other countries (30) including China (21) and the United States (31, 32). Potentially, chronic inflammation, oxidative stress, and cerebrovascular injury can all be brought on by hypertension leading to cognitive decline. Moreover, high blood pressure for long periods may increase the chance of small vessel damage in the brain which has been linked with dementia. Hypertension itself has a direct negative effect on cerebral vasoreactivity (33, 34). Through mechanisms unrelated to clinical stroke, high blood pressure increases the risk of dementia and cognitive decline. It has also a detrimental influence on cognition throughout life, raising the risk of both early-onset and late-life dementia (35, 36).

### Strengths and limitations of the study

Important strengths of our study were that it was multicentered, the response rate was 100% which was achieved through informing the participants about the relevance of the topic, data collection was done by a treating psychiatrist, the result of the cognitive assessment was informed for each patient, and rehabilitation team used the feedback to approach the patients. Moreover, the diagnosis of stroke was confirmed using a CT scan. The longitudinal nature of the study also is an additional strength to identify the predictors at 3 months post-stroke. Our study included patients who were admitted to the hospital whereby stroke severity extremes might not be included. The result of this study may be limited as it does not include pre-stroke cognitive status of the participants. An extended follow-up with baseline and follow-up cognitive outcome would be ideal to understand the progression of PSCI in the long run.

### Implications of the study

PSCI incidence is high among stroke survivors in Ethiopia and needs attention during stroke acute management and rehabilitation. In Sub-Saharan Africa, clinical assessments such as PSCI may provide additional prognostic evidence especially when advanced investigations such as CT scans are unavailable. Further research in this space is needed in low-and middle-income countries to advance stroke management in these countries.

## Conclusion

In the current study, nearly one-third of stroke survivors developed PSCI. Furthermore, we identified PSCI as being longitudinally associated with age, sex, and admission systolic blood pressure, mRS, and GCS scores. Moreover, further research is needed with a larger sample size, showing a time trend and extended follow-up time.

## Data availability statement

The raw data supporting the conclusions of this article will be made available by the authors, without undue reservation.

## Ethics statement

The studies involving human participants were reviewed and approved by Ethical review committee of College of Health science, Debre Tabor University. The patients/participants provided their written informed consent to participate in this study.

## Author contributions

GA, GY, and FA: conceptualization and formal analysis. GA and GY: data curation. GA: funding acquisition and supervision. GA, AA, and ATA: investigation. GA, SS, and DA: methodology. GA and DA: project administration. GA, DA, and AA: resources. GA, DA, and ATA: software. GA, DA, and SS: validation. GA, AA, and SS: visualization. GA, FA, GY, and SS: writing – original draft. GA and SS: writing – review and editing. All authors contributed to the article and approved the submitted version.

## Conflict of interest

The authors declare that the research was conducted in the absence of any commercial or financial relationships that could be construed as a potential conflict of interest.

## Publisher’s note

All claims expressed in this article are solely those of the authors and do not necessarily represent those of their affiliated organizations, or those of the publisher, the editors and the reviewers. Any product that may be evaluated in this article, or claim that may be made by its manufacturer, is not guaranteed or endorsed by the publisher.
